# Basic emotions reported by individuals with persistent physical symptoms receiving exposure therapy versus healthy lifestyle promotion in primary care

**DOI:** 10.1038/s41598-026-39962-x

**Published:** 2026-02-17

**Authors:** Jonna Hybelius, Sandra af Winklerfelt Hammarberg, Sigrid Salomonsson, Caroline Wachtler, Majken Epstein, Anna Olsson, Emma Strand, Lina Söderström Winter, Alice Ahnlund Hoffmann, Edward Spansk, Tomas Åkerlund, Daniel Björkander, Amanda Kosic, Gabriel Chahin, John Wallert, Eva Toth-Pal, Steven Nordin, Michael Witthöft, Erland Axelsson

**Affiliations:** 1 Division of Family Medicine and Primary Care, Department of Neurobiology, Care Sciences and Society, Karolinska Institutet, Huddinge, Sweden; 2 Liljeholmen University Primary Health Care Center, Academic Primary Health Care Center, Region Stockholm, Stockholm, Sweden; 3 Centre for Psychiatry Research, Department of Clinical Neuroscience, Karolinska Institutet, & Stockholm Health Care Services, Region Stockholm, Stockholm, Sweden; 4 Division of Psychology, Department of Clinical Neuroscience, Karolinska Institutet, Stockholm, Sweden; 5 Meliva Primary Health Care Center Kungshörnet, Uppsala, Sweden; 6 School of Law, Psychology and Social Work, Örebro University, Örebro, Sweden; 7 Department of Psychology, Umeå University, Umeå, Sweden; 8 Department of Clinical Psychology and Psychotherapy, Ruhr University Bochum, Bochum, Germany

**Keywords:** Persistent physical symptoms, Emotions, Primary care, Internet-delivered, Exposure therapy, Healthy lifestyle promotion, Health care, Psychology, Psychology

## Abstract

The importance of fear has been emphasized in research and treatment focusing on persistent physical symptoms (PPS). Recognizing a broader range of emotions may inform theory and personalized care. This study aimed to examine what basic emotions individuals with PPS experience related to their physical symptoms, the correlations between such emotions and overall somatic symptom burden and disability, and whether emotions change with treatment. Analyses drew on data from a randomized controlled trial that compared internet-delivered exposure therapy with healthy lifestyle promotion for patients with PPS (n = 159), supplemented by data from age- and gender-matched healthy volunteers (n = 160). Participants self-reported emotions related to their physical symptoms. Mean differences were evaluated within a linear regression modeling framework. Compared with the healthy volunteers, PPS participants scored significantly higher on anger, disgust, fear, sadness, and shame, and lower on joy. Disgust, fear, sadness, and shame correlated significantly with somatic symptom burden; anger, fear, sadness, and shame with disability. All negatively valenced emotions reduced significantly in both interventions, without significant between-group effects. Joy increased significantly in exposure. These results highlight the potential relevance of diverse emotions in PPS. To facilitate personalized care, future work could evaluate the unique contribution of specific emotions to clinical outcomes.

**Supplementary Information:**

The online version contains supplementary material available at 10.1038/s41598-026-39962-x.

## Introduction

Persistent physical symptoms (PPS)—somatic complaints that last several months or more—are prevalent across all healthcare settings and associated with substantial suffering and costs^1^. Within behavioral medicine, theoretical approaches to PPS have often emphasized the role of fear, with the fear-avoidance model of pain holding a formative position^2^. According to this model, the fear of pain and the avoidance of pain-related stimuli, such as physical activity, predict chronicity. This relationship may stem from several mechanisms. Heightened physiological arousal may be interpreted as bodily symptoms, rather than recognized as part of the experience of negative affect^3^. Fear may also drive hypervigilance and dysfunctional behavioral strategies, such as body checking and avoidance^4^. This may lead to physical inactivity and a restrictive behavioral repertoire focused on minimizing bodily distress, thereby exacerbating symptoms and long-term functional impairment.

Treatments targeting fear and avoidance have been found promising for chronic pain and other domains of PPS, including functional somatic syndromes such as irritable bowel syndrome, and persistent symptoms related to somatic diseases like asthma or atrial fibrillation^5–7^. However, many patients with PPS do not experience sufficient benefit from existing clinical interventions, fear-focused or otherwise. This was illustrated by a review of European longitudinal studies of patients treated for PPS, which indicated symptom persistence or deterioration in 62% of studies based in primary care, and in 56% of studies in secondary care^8^.

Looking more broadly at human functioning, fear represents just one example of a universal and distinct emotion, with other common examples including anger, disgust, joy, sadness, and surprise^9^. Shame, characteristic of the stigma commonly reported by patients with PPS, has also been considered for inclusion alongside the previously mentioned “big six”. It is possible that fear may not play as unique a role in the perception of somatic complaints as implied by the literature’s emphasis on this emotion. For example, empirical work on the Hierarchical Taxonomy of Psychopathology (HiTOP) has indicated that distress related to physical symptoms appears to be subsumed under an overarching emotion dysregulation superspectrum^10^. This superspectrum encompasses the higher-order dimension of negative affect, which includes facets such as emotional lability, anger/irritability, and rumination, thereby highlighting the potential role of emotion regulation difficulties and negatively valenced emotional responses more broadly in distress related to somatic complaints. Consistent with this, experimental findings using a sadness induction paradigm have demonstrated elevated levels of dysfunctional emotion regulation strategies, such as rumination, among participants with PPS (i.e., DSM-5 somatic symptom disorder) as compared to healthy controls^11^. Studies have also indicated associations between elevated anger, dysfunctional expression of anger, and physical symptom burden in individuals with chronic pain and persistent symptoms with unclear etiology^12,13^. Although research is limited, some empirical findings are also suggestive of various emotions playing a role in patient groups with clearly defined somatic conditions. For instance, elevated anger has been linked to momentary worsening of symptoms in asthma and rheumatoid arthritis^14^. Further, cross-sectional findings indicate elevated self-disgust—internalized disgust directed towards oneself—among patients with dermatological diseases^15^. There is also experimental evidence that manipulating gastric rhythm affects disgust avoidance in healthy individuals, suggesting a causal role for gastric dysrhythmias in disgust-related avoidance^16^. In summary, there is reason to suspect that PPS could be associated with other emotions in addition to fear.

**Fig. 1:**
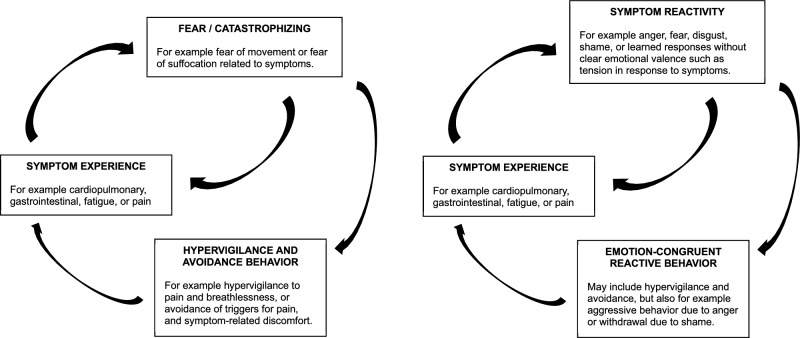
Models of symptom reactivity. Left: Mainstream fear-avoidance model of persistent physical symptoms. The experience of physical symptoms is exacerbated by physiological changes inherent to fear, top-down processes where fear implies the expectation of symptoms, excessive attention to symptoms and related phenomena (hypervigilance), and avoidance behavior which precludes useful experiences, maintains fear over time, and implies a restrictive behavior repertoire where symptoms become a central part of life. Right: Broadened model to encompass a wider spectrum of emotional responses, and responses without clear emotional valence, to physical symptoms. The experience of physical symptoms is exacerbated by physiological changes inherent to emotions, top-down processes where negatively valenced emotions imply the expectation of symptoms, excessive attention to symptoms and related phenomena (hypervigilance), and various emotion-congruent reactive behaviors that preclude useful experiences, maintains dysfunctional responses over time, and implies a restrictive behavior repertoire where symptoms become a central part of life.

This study adopted a broad perspective on basic emotions related to PPS and aimed to evaluate its relevance for patients treated in primary care. As illustrated in Fig. 1, while a traditional fear-avoidance model emphasizes how fear drives physiological arousal, hypervigilance toward symptoms, avoidance behavior, and a restrictive behavioral repertoire, we adopted a wider theoretical framework. This model also considers the relevance of other emotions and learned responses for changes in physiology, altered perception, excessive emotion-congruent behavior, and diminished quality of life.

For this study, we hypothesized that (i) compared with healthy volunteers, clinical trial participants with PPS would score higher on all emotions with a negative valence (anger, disgust, fear, sadness, shame) related to somatic symptoms, and that (ii) there would be reductions in all negatively valenced emotions during exposure therapy and healthy lifestyle promotion. Building on mainstream work on behavioral avoidance, we also expected (iii) exposure therapy to have a larger effect than healthy lifestyle promotion on fear, and that (iv) higher pre-treatment fear would be predictive of a larger reduction in fear and (v) a larger advantage of exposure therapy relative to healthy lifestyle promotion.

## Methods

### Design

This was a secondary study based on the SOMEX1 trial: a randomized controlled trial that compared exposure therapy to healthy lifestyle promotion for individuals with PPS (N = 161) at Liljeholmen University Primary Health Care Center, in collaboration with the Department of Neurobiology, Care Sciences and Society, Karolinska Institutet, Stockholm, Sweden^17^. Because two participants had missing data on the basic emotions questionnaire, the number analyzed in this study was 159. Healthy volunteers (N = 160) were also recruited for psychometric analyses and to evaluate the relevance of clinical findings. This study was approved by the Swedish Ethical Review Authority (2021-01,400, 2023-06,574-01), and all aspects of the methodology complied with the Declaration of Helsinki. All participants provided informed consent as a prerequisite for enrollment. The study was preregistered at ClinicalTrials.gov (NCT04942028, 17/06/2021), and the statistical analysis plan was preregistered with the Open Science Framework (osf.io/rgq6t).

### Recruitment

For the clinical trial, we recruited adults with distress related to PPS via primary care clinics and social media advertisements^17^. Applicants underwent an interview with a mental health professional to survey clinical information and eligibility. The interview included the Mini-International Neuropsychiatric Interview and the Health Preoccupation Diagnostic Interview. Participants could endorse any symptom domain, including cardiopulmonary symptoms, fatigue, gastrointestinal complaints, or pain. Symptoms could be of any origin, including somatic diseases such as asthma or inflammatory bowel disease, be related to a functional somatic syndrome such as fibromyalgia or irritable bowel syndrome, or lack a medical attribution. For inclusion, participants had to be either “much bothered” by at least one physical symptom (item of the Patient Health Questionnaire 15 [PHQ-15]) or report a moderate somatic symptom burden (PHQ-15 ≥ 10), with somatic symptoms having been present for a minimum of 4 months. Similar to the bodily distress disorder diagnosis of the International Classification of Diseases 11, the dominant clinical problem was required not to be best explained as primary pathological health anxiety or a non-somatoform psychiatric disorder. Participants had to live in Stockholm, be fluent in Swedish, and not suffer from a serious psychiatric disorder or severe suicidal ideation. There could not be other medical obstacles to participation, such as alarm symptoms warranting investigation or conditions making physical activity potentially harmful. Continuous psychotropic medication had to be non-existent or stable for ≥ 4 weeks. Alcohol or substance use could not risk interfering with treatment, and participants could not have planned absence > 1 week during treatment.

We recruited healthy volunteers via online advertisement on social media. Participants were age- and gender-matched to the clinical sample and underwent a structured telephone interview analogous to that for the clinical applicants. Volunteers received a gift card valued at 300 SEK as compensation. They had to be ≥ 18 years old and familiar with the Swedish language. Exclusion criteria were a psychiatric disorder endorsed during application or the interview, or a score of 2 (*“bothered a lot”*) on any item of the PHQ-15.

### Outcomes

#### Overview of measurement strategy

All participants completed self-report questionnaires via an online measurement platform (*“BASS”*). The PPS participants completed an application screening (only used to estimate test–retest reliability), a baseline assessment (pre-treatment), 9 weekly assessments during treatment, and a post-treatment assessment (week 10). The healthy volunteers completed an application screening (baseline assessment) and a second assessment 2 weeks later.

#### Emotions experienced in relation to physical symptoms

We assessed basic emotions related to physical symptoms during the past week using a new study-specific questionnaire. A list comprising examples of common physical symptoms was given, followed by the prompt *“How have you felt about your bodily symptoms during the past week?”*. Respondents rated to what extent they had experienced anger, disgust, fear, joy, sadness, shame, and surprise (7 items) related to their physical symptoms. To facilitate participants’ differentiation between emotions, all items were presented simultaneously as a list. For each emotion, a response could be indicated on a visual-analogue scale (VAS). For example, the item concerning anger read *“Anger: Your symptoms made you angry”*, with a VAS scored from *“No, not correct at all”* to *“Yes, completely correct”*. Each VAS was scored 0–10. The full instrument is provided in the Supplement.

#### Somatic symptom burden, disability, and psychiatric symptoms

Participants completed the PHQ-15 as a measure of somatic symptom burden, the 12-item self-report World Health Organization Disability Assessment Schedule (WD2-12) as a measure of disability, the GAD-7 as a measure of general anxiety, the 14-item Health Anxiety Questionnaire (HAI-14) as a measure of health anxiety, and the Patient Health Questionnaire 9 (PHQ-9) as a measure of depressive symptoms.

### Clinical trial randomization and treatments

Participants in the clinical trial were randomized (1:1) to 10 weeks of exposure therapy or healthy lifestyle promotion. Both treatments were delivered in a therapist-guided, internet-based format, through text-based modules resembling book chapters. Communication took place via an email-like system. In exposure therapy, the treatment rationale conveyed that heightened reactivity to physical symptoms, such as unwanted emotions and changes in attention, likely contributes to a higher subjective somatic symptom burden. To repeatedly engage in activities that evoke symptoms or associated distress in a structured manner (exposure), while refraining from behavioral strategies aimed at short-term relief (response-prevention), was framed as a method for achieving long-term improvement in symptoms and well-being. The exposure therapy addressed fear and anxiety contingent on physical symptoms, but also encouraged participants to work with other emotional responses to somatic complaints. Therapists were encouraged to investigate participants’ emotional responses to symptoms broadly and provide support in tailoring exposure exercises to approach various negatively valenced emotions. In healthy lifestyle promotion, participants were encouraged to formulate and adhere to a structured plan for improved lifestyle behaviors to achieve enhanced overall mental and physical health, including beneficial effects on somatic complaints. The protocol was developed for the clinical trial, with content aligning with information provided via the national Swedish healthcare information platform. Further details on the randomization procedure and interventions are provided in the primary publication^17^. The exposure protocol is available from the Open Science Framework (osf.io/cnbwj).

### Statistical analysis

Analyses were conducted in R 4.3.1 and Stata 18.0 per an openly registered plan (osf.io/rgq6t). We evaluated the test–retest reliability of the basic emotions questionnaire over the two measurement points among the healthy volunteers and using data from a subset of PPS participants (n = 20) who completed their pre-treatment assessment within 14 days from screening (reported as Pearson correlations [*r*], Spearman correlations [*ρ*], and two-way random effects absolute agreement intraclass correlation coefficients [ICC]). Because no directed hypotheses were pre-registered for the analyses of (i) correlations versus disability, (ii) between-group effects on emotions except for fear, or (iii) within- and between-group moderator analyses except concerning fear, we controlled the false discovery rate of these tests using the Benjamini–Hochberg procedure. Mean differences in emotions at baseline were tested using 95% bias-corrected and accelerated confidence intervals based on 1000 bootstrap replications. In the PPS sample, Pearson correlations were calculated between emotions, somatic symptom burden, and disability at baseline. Linear mixed effects regression models were fitted to study change in each emotion over the pre- to post-treatment phase of the trial. Predictors were time (pre-post), condition (1/0), the time × condition interaction. The models included a random intercept. Within- and between-group effects were tested. In analogous models, somatic symptom burden and disability were modelled as outcomes, and emotions (baseline values) were evaluated as within- and between-group moderators. Models of somatic symptom burden (measured weekly) also included a random slope (time, within participants). Missing data was multiply imputed using chained equations in mice 3.16.0. Although the clinical trial was powered for its primary outcome^17^, we estimate that power for most tests in this secondary study was 80% or higher to study moderate to large, but not small, effects. Standardized effects (Cohen’s *d*) were calculated by dividing model-implied means by the pre-treatment standard deviation (non-imputed data) of the full clinical sample for within-group comparisons, and by the endpoint standard deviation (non-imputed data) for between-group tests.

## Results

### Sample characteristics

Sample characteristics are reported in Table 1. The average participant in both samples was a 48-year-old female with a postsecondary education, recruited via social media. While the average PPS participant reported a moderate somatic symptom burden, healthy volunteers reported minimal symptoms. PPS participants endorsed a variety of diagnoses and comorbidities. The most frequently reported functional somatic syndromes were irritable bowel syndrome (57/159, 36%) and fibromyalgia (21/159, 13%). Although not always the focus of the intervention, the most frequently reported somatic diseases were osteoarthritis (48/159, 30%), hypertension (39/159; 25%), and asthma (33/159; 21%).

**Table 1:** Baseline characteristics in patients with persistent physical symptoms (n = 159) and healthy volunteers (n = 160).

	PPS patients	Healthy participants					
	(*n* = 159)	(*n* = 160)					
Sociodemographics							
Age, Mean (SD), range	48.1 (12.1), 20–73	47.8 (12.6), 18–75					
Female, *n* (%)	135 (85%)	136 (85%)					
Postsecondary education, *n* (%)	129 (81%)	138 (86%)					
Employed, *n* (%)	126 (79%)	121 (76%)					
Married or de facto, *n* (%)	108 (68%)	117 (73%)					
Clinical characteristics (relevant for patients only)							
PPS duration in years, Mean (SD), range	11.7 (10.8), 0.5–57	–					
DSM-5 psychiatric disorders, *n* (%)							
Somatic symptom disorder	68 (43%)	–					
Major depressive disorder	37 (23%)	–					
At least one anxiety disorder^a^	45 (28%)	–					
Conventional symptom scales, Mean (SD), median (range)							
Somatic symptom burden (PHQ-15)	12.9 (4.7), 13 (2–30)	2.5 (2.3), 2 (0–12)					
General anxiety (GAD-7)	6.0 (4.3), 5 (0–20)	1.0 (1.7), 0 (0–9)					
Depression core symptoms (PHQ-2)	1.6 (1.5), 1 (0–6)	0.2 (0.6), 0 (0–4)					
Disability (WD2-12)	25.1 (16.2), 23 (0–68.8)	2.1 (4.9), 0 (0–31.3)					

	PPS patients (n = 159)	Healthy participants (n = 160)	Bootstrapped mean differences			PPS patients (n = 159)	
	M *(SD)*, median (range)	M *(SD)*, median (range)	MD, BCa 95% CI	Adj. R-squared	*d*	*r*	*r*
						PHQ-15	WD2-12
Basic emotions^b^							
Anger	4.2 (3.2), 3.8 (0–10)	0.6 (1.1), 0.2 (0–6.4)	3.54 (3.03, 4.09)	0.355	1.48	0.12	0.26***
Disgust	1.9 (2.9), 0.3 (0–9.8)	0.4 (1.1), 0.2 (0–10)	1.49 (1.01, 1.96)	0.099	0.67	0.18*	0.10
Fear	3.0 (2.9), 2.2 (0–10)	0.6 (1.0), 0.2 (0–6.1)	2.41 (1.98, 2.91)	0.233	1.11	0.17*	0.26***
Joy	0.3 (0.6), 0.1 (0–3.9)	0.5 (1.4), 0.1 (0–10)	-0.23 (-0.56, -0.04)	0.009	-0.22	0.10	-0.00
Sadness	6.1 (3.2), 6.8 (0–10)	0.9 (1.4), 0.3 (0–7.5)	5.26 (4.68, 5.78)	0.532	2.13	0.25**	0.43***
Shame	3.3 (3.2), 2.3 (0–10)	0.6 (1.3), 0.2 (0–9.7)	2.67 (2.16, 3.20)	0.228	1.09	0.20*	0.23***

Mean differences were estimated using 1000 bootstrap replications. Tests of Pearson correlations between basic emotions and the WD2-12 were adjusted for multiple testing. Correlations with the PHQ-15 were not adjusted for multiple testing due to pre-specified specific hypotheses of a positive correlation with each emotion with a negative valence. All scales were administered as self-report questionnaires via the Internet at the baseline assessment. PHQ-15, Patient Health Questionnaire 15 (conventional 4-week version; scored 0–30); PPS, persistent physical symptoms; WD2-12, 12-item self-report World Health Organization Disability Assessment Schedule 2 (scored 0–100).

^a^Not including obsessive–compulsive disorder or post-traumatic stress disorder.

^b^Study-specific questionnaire surveying the experience of anger, disgust, fear, joy, sadness, shame, and surprise related to physical symptoms over the past week.

* *p* < 0.05.

** *p* < 0.01.

*** Significance determined after adjusting for multiple testing.

### Missing data and adherence to the protocol

The PPS participants had no missing data at screening or baseline, and the post-treatment assessment was completed by 153/159 (96%). The healthy volunteers had no missing data. Among PPS participants, exposure therapy was completed (initiated ≥ 5 of 10 modules) by 67% (53/79) and healthy lifestyle promotion by 81% (65/80).

### Test–retest reliability and construct validity of emotions questionnaire

In the healthy volunteer data, test–retest reliability was poor, both for the study-specific emotions questionnaire (over a mean of 14.7 days [SD = 0.9]; *r*s = 0.20–0.59 across emotions, *ρ* = 0.32–0.49, ICCs = 0.33–0.73) and previously validated measures of anxiety (e.g., GAD-7; *r* = 0.52, *ρ* = 0.56, ICC = 0.52) and depression (PHQ-2; *r* = 0.56, *ρ* = 0.35, ICC = 0.56).

In the PPS participant data, the test–retest reliability for the emotions was acceptable (over a mean of 6.5 days [SD = 3.6]; *rs* = 0.60–0.88, *ρs* = 0.46–0.83, ICCs = 0.68–0.81), with the exception of surprise (*r* = 0.07, *ρ* = 0.45, ICC = 0.60). We therefore refrained from further analysis of the surprise item, although the interpretation of these findings is complicated by the possibility that low test–retest reliability could partly reflect that surprise may be inherently less stable across time points than the other emotions. Correlations with previously validated questionnaires indicated adequate construct validity (*r*s = 0.34–0.41) (Supplementary Tables DS1–DS3 for details).

Due to the limited reliability of the healthy volunteer data, these were only used for an overview of distributions in emotions, and also tests of mean differences versus the clinical sample at baseline, because on these outcomes we expected mostly large effects. We refrained from using the healthy volunteer data for other planned inferential analyses. A consequence of this was that, based on the PPS participant data alone, there was no longer sufficient statistical power to conduct inferential tests on the basis of multivariable models to evaluate whether each specific emotion explained a unique proportion of the variance in somatic symptom burden and disability.

### Distribution of basic emotions related to physical symptoms

**Fig. 2:**
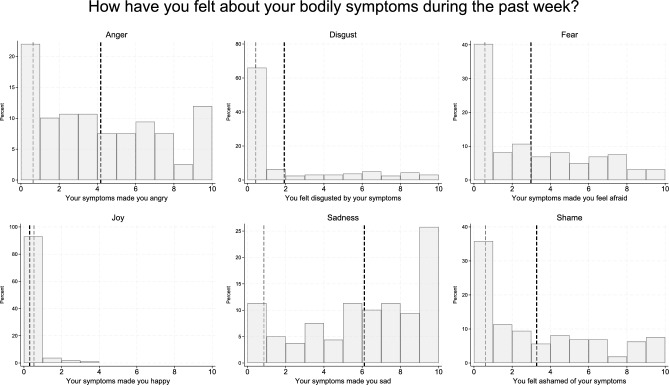
Distributions of basic emotions. Distributions of basic emotions reported in relation to physical symptoms (visual analogue scale from 0 “No, not correct at all” to 10 “Yes, completely correct”) at baseline among clinical trial participants with persistent physical symptoms (PPS group; n = 159). The bold dashed lines represent the means for the PPS group, and the thin dashed lines represent the means for the healthy volunteers (n = 160).

The distribution of emotions related to physical symptoms reported by PPS participants and healthy volunteers is illustrated in Table 1 and Fig. 2. The PPS subsample scored significantly higher on all negatively valenced emotions (*d*s = 0.67–2.13) and lower on joy (*d* =  − 0.22) (Table 1).

### Correlations between basic emotions, somatic symptom burden, and disability

Baseline correlations between emotions related to physical symptoms, somatic symptom burden, and disability are reported in Table 1. Somatic symptom burden exhibited weak but significant positive correlations with disgust, fear, sadness, and shame (*r*s = 0.17–0.25). Disability exhibited a moderate and significant positive correlation with sadness (*r* = 0.43), and weak but significant positive correlations with anger, fear, and shame (*r*s = 0.23–0.26). Supporting the analysis of these basic emotions as distinct phenomena, all Pearson correlations between the basic emotions were below 0.70 (*r*s = 0.24–0.66; Table DS4). Secondary, exploratory analyses of the correlations between basic emotions and the symptom domain-specific subscales of the PHQ-15 are reported in the Supplement (Table DS5).

### Effect of exposure therapy and healthy lifestyle promotion on basic emotions

In both exposure therapy and healthy lifestyle promotion, significant small to moderate within-group reductions were seen in all negatively valenced emotions (*d*s = 0.22–0.71; Table 2). Additionally, in exposure therapy, there was a moderate and statistically significant increase in joy (*d* = -0.73). There were no significant differences between exposure therapy and healthy lifestyle promotion in their effects on the basic emotions (Table 2).

**Table 2:** Mean change in basic emotions related to somatic symptoms in internet-delivered exposure therapy versus healthy lifestyle promotion over 10 weeks.

Outcome	Treatment	Change from pre-treatment to post-treatment					
		Within-group change			Difference in change		
		est (95% CI)	*d*	*p*	est (95% CI)	*d*	*p*
Anger	Exposure	− 1.23 (-1.91, -0.55)	0.39	0.000***	0.42 (-0.54, 1.37)	-0.15	0.389
	HLP	-1.64 (-2.32, -0.97)	0.52	0.000***			
Disgust	Exposure	-0.75 (-1.25, -0.26)	0.26	0.003**	-0.11 (-0.80, 0.59)	0.05	0.760
	HLP	-0.65 (-1.13, -0.16)	0.22	0.009**			
Fear	Exposure	-0.90 (-1.44, -0.36)	0.31	0.001**	-0.09 (0.85, 0.66)	0.04	0.809
	HLP	-0.80 (-1.33, -0.28)	0.28	0.003**			
Joy	Exposure	0.41 (0.07, 0.74)	-0.73	0.019*	0.15 (-0.31, 0.61)	-0.11	0.523
	HLP	0.25 (-0.07, 0.58)	-0.46	0.120			
Sadness	Exposure	-2.29 (-3.01, -1.58)	0.71	0.000***	-1.30 (-2.30, -0.30)	0.39	0.011^a^
	HLP	-1.00 (-1.69, -0.30)	0.31	0.005**			
Shame	Exposure	− 1.30 (− 1.93, − 0.68)	0.41	0.000***	− 0.45 (− 1.33, 0.42)	0.16	0.311
	HLP	− 0.85 (− 1.47, − 0.24)	0.27	0.007**			

Estimates derived from linear mixed effects regression models fitted on mulitply imputed data. The study specific basic emotions questionnaire was administered as a self-report questionnaire via the Internet at the pre-treatment (pre) and post-treatment assessment (post). Standardized within-group effects (*d*s) were calculated as the negated point estimate for the model-implied change (as listed under “est (95% CI)”), divided by the pre-treatment standard deviation for the entire sample in the non-imputed data. Standardized between-group effects (*d*s) were calculated as the point estimate for the model-implied difference (as listed under “est (95% CI)”), divided by the endpoint standard deviation for the entire sample in the non-imputed data. Tests of within-group effects not adjusted for multiple testing due to pre-specified specific hypotheses of reductions in each emotion with a negative valence. HLP, healthy lifestyle promotion.

^a^ Not significant after adjusting for multiple testing.

* *p* < 0.05, ** *p* < 0.01, *** *p* < 0.001.

### Basic emotions related to physical symptoms as potential effect moderators

A higher pre-treatment level of fear related to physical symptoms was predictive of a larger within-group reduction of fear in exposure therapy (*b* =  − 0.48 [− 0.64 to − 0.32]; *p* < 0.000) and in healthy lifestyle promotion (*b* =  − 0.50 [− 0.65 to − 0.35]; *p* < 0.000). Pre-treatment fear related to physical symptoms did not moderate the effect of exposure therapy relative to healthy lifestyle promotion on fear. After adjustment for multiple testing, none of the basic emotions were significant moderators of the within-group reduction, or between-group effect, on somatic symptom burden or disability (Supplementary Table DS6).

## Discussion

This study presented a uniquely broad overview of basic emotions experienced in relation to persistent physical symptoms by a heterogeneous sample treated in primary care. At baseline, PPS participants reported experiencing not only fear, but also anger, disgust, sadness, and shame related to their physical symptoms, whereas ratings of negatively valenced emotions related to physical symptoms among healthy volunteers were close to zero. In the PPS sample, significant correlations with somatic symptom burden and disability were seen for a variety of negatively valenced emotions. Over the course of both exposure therapy and healthy lifestyle promotion, significant reductions in all negatively valenced emotions were observed. Contrary to expectation, exposure therapy did not exhibit a larger effect on fear relative to healthy lifestyle promotion. A higher pre-treatment level of fear related to physical symptoms was, however, predictive of a larger fear reduction in both treatments. None of the basic emotions significantly moderated the effects on somatic symptom burden or disability. In summary, our hypotheses of a broad set of negatively valenced emotions being overrepresented in individuals with PPS, all of these emotions being reduced with therapy, and a higher level of fear being predictive of a larger reduction in fear, were all corroborated. In contrast, our expectations of a specific benefit of exposure therapy for the reduction of fear, and fear being a moderator of other outcomes, were not supported. Taken together, these results illustrate the potential relevance of a broad spectrum of emotions in persistent physical symptoms.

### Comparison with prior research on specific emotions

To our knowledge, no previous studies have examined how a broad set of emotions experienced related to physical symptoms are distributed and change from pre- to post-treatment in a diverse clinical sample with PPS. Still, our findings align with several strains of the existing literature. In this study, PPS participants reported anger related to their somatic complaints, and levels of anger decreased with treatment (exposure *d* = 0.39; HLP *d* = 0.52). This dovetails, for example, with previous evidence of elevated anger among participants with chronic headache as compared to healthy controls and experimental findings indicating that an anger expression intervention can improve pain control in chronic pain patients^18,19^. Further, elevated baseline disgust related to physical symptoms was seen among PPS participants, tentatively aligning with previous studies suggesting that disgust propensity and sensitivity predict hypervigilance in non-clinical participants^20^ and correlate with IBS-related quality of life^21^. The improvements seen in disgust over treatment are also consistent with previous findings suggesting that cognitive behavioral therapy encompassing exposure can improve disgust propensity in obsessive–compulsive disorder^22^.

Contrary to expectation, the reduction in fear during exposure therapy was small (*d* = 0.31), without superiority over healthy lifestyle promotion. Moreover, fear related to physical symptoms was not significantly correlated with pain (subscale of the PHQ-15; Supplementary Table DS5). Taken together, these results are surprising, given the mainstream view of exposure as an intervention primarily concerned with fear reduction and increased fear toleration. Notably, in the present study, the largest within-group effects in exposure therapy were instead seen for increased joy (*d* = -0.73) and decreased sadness (*d* = 0.71). These observations resonate with preliminary findings showing that treatment aimed at increasing happiness can be beneficial for chronic pain (within-group *d* = 1.08 for pain intensity [conservatively estimated])^23^ and replicated findings of an association between experimentally induced sadness and symptom reporting [e.g.^24^]. PPS participants also acknowledged shame related to their symptoms, consistent with previous studies in which shame has been linked to psychiatric disorders centered around the perception of the body [e.g.^25^] and to avoidance behaviors^26^. It would be premature to draw far-reaching conclusions from the preliminary findings of this study, given that the emotions questionnaire has not been previously validated. However, the fact that fear did not stand out as more central than other basic emotions related to persistent symptoms in this study can be seen as indicative of the potential benefits of studying and targeting a broader scope of emotions in this patient group.

### Expanding the view of emotions in persistent physical symptoms

For decades, much of the theoretical and empirical work on PPS, especially in applied clinical settings, has focused on the role of avoidance and fear^2,4^. A mainstream cognitive-behavioral conceptualization of factors contributing to chronicity sketches out a sort of “symptom phobia”, where catastrophizing is associated with heightened reactivity and seen as the motivator for hypervigilance and behavioral avoidance, with adverse long-term repercussions. This is a successful paradigm that has led to many fruitful developments. The findings presented here, however, broaden the aforementioned view. For example, anger may drive rumination and heightened attention to physical symptoms^27^, just as shame may drive social isolation and avoidance^28^. Both anger and shame also imply heightened physiological arousal, and thereby potentially somatic symptoms. A problem with many studies of treatments for PPS is that fear or anxiety are the only emotional responses investigated, leaving no room for the alternative hypothesis of effects being related to other emotional responses, or more general deviations in emotional processing. Evidence in support of a broader perspective on emotions in PPS can be found without much effort, but has not always bled into the cognitive-behavioral literature. A noteworthy example is the literature on stigma, characterized by the experience of shame, which is well-documented in various subgroups with PPS^29^.

### Better to focus on specific basic emotions or general emotional dysfunction?

An important question for future research is when and to what degree it is relevant to focus on specific basic emotions (e.g., anger, disgust, fear, sadness, shame) as opposed to more general measures of reactivity and emotional dysfunction in relation to persistent physical symptoms. Although our results appear to indicate that fear is not the only basic emotion of relevance for persistent physical symptoms, the poor reliability of the healthy volunteer data (see above) made it impossible to run multivariable models with sufficiently powered tests to evaluate whether specific emotions contributed unique and meaningful explanatory value. This implies that, for example, the association of somatic symptom burden with disgust, fear, sadness, and shame (as reported in Table 1) could potentially reflect that all these presumably specific emotion measures capture the same general pattern of emotional dysfunction. In further advancing this field, arguments for continued research focusing on specific emotions include standard arguments for the distinctness of emotions—such as recognition across ages and cultures, and partly distinct physiology and functions—and that certain specific emotions—primarily fear—are already widely acknowledged to play a particularly important role for many patients. Arguments for focusing instead on general emotional dysfunction include simplicity (ontological parsimony), statistical considerations such as fewer inferential tests in analyses, and the fact that at least some presumed mechanisms—notably, physiological arousal leading to heightened somatic symptom burden, thereby prompting further unwanted responses—could be relevant regardless of emotional valence. Both “splitting” and “lumping” views on reactivity to persistent physical symptoms are compatible with the HiTOP paradigm, which has been suggestive of a close connection between somatic symptom distress and a broad spectrum of internalizing psychopathology^10^. Both views are also compatible with the predictive processing model of PPS, which emphasizes the role of negative emotions in the emergence and maintenance of PPS^30^. Psychotherapies aimed at improving general emotion regulation have been found effective for these patients[e.g.^31^], further highlighting the potential benefits of a broad perspective on emotions; regardless of whether this implies measuring several specific emotions or focusing on broader measures of reactivity to somatic symptoms. We encourage future work in this field to extend beyond an exclusive focus on fear.

### Limitations

The primary limitation of this study was the use of a newly developed, not previously validated, questionnaire to measure emotions related to physical symptoms. This emotions questionnaire had poor reliability in the healthy volunteer data, probably due to a lack of variance. The questionnaire did, however, exhibit adequate test–retest reliability and correlations indicative of construct validity in the PPS sample. Another limitation was that the healthy volunteers were self-referred with minimal psychiatric and somatic symptoms, which means that differences versus the PPS sample were larger than what would be expected in a comparison between the PPS sample and the general population. A threat to internal validity was that individuals with PPS may be prone to exhibit difficulties in recognizing and labeling emotional experiences (alexithymia), and therefore not report their own emotional states in a reliable manner. Findings in this regard are, however, mixed^32^. Furthermore, as was also pointed out above, because no multivariable analyses were conducted, it was not possible to determine which emotions that explained a unique proportion of variance in symptom burden.

### Clinical and research implications

This study illustrates the potential clinical benefits of considering emotional responses to physical symptoms beyond fear and avoidance. To facilitate personalized care, treatment for PPS may be tailored not only to physical symptom domains but also to different emotional responses to symptoms, depending on the clinical presentation of the individual patient.

Future research should examine which emotions may exert unique effects on symptom burden, symptom preoccupation, and disability in patients with PPS. Additionally, studies could investigate whether other emotions, in addition to fear, predict long-term outcomes in PPS. It could also be informative to further investigate the relationship between the experience of various emotions and physical symptoms in experimental paradigms. Lastly, it would be desirable for future studies evaluating interventions for PPS to investigate changes across a broad spectrum of emotions related to somatic complaints.

## Conclusions

Patients with persistent physical symptoms experience a broad spectrum of negatively valenced emotions. More than fear, patients also report anger, disgust, sadness, shame, and low joy related to their physical symptoms. Although their unique contributions to effects are unclear, several of these emotions were found to be associated with symptom severity and disability in this study. Further, all negatively valenced emotions changed with exposure therapy and healthy lifestyle promotion. This indicates potential benefits of focusing on a broader set of emotions associated with persistent physical symptoms to further improve research and care for this heterogeneous group.

## Supplementary Information

Supplementary Information.

## Data Availability

The datasets analyzed during the current study are not publicly available at present. This is because individual participant data (IPD) cannot currently be shared due to restrictions under Swedish and EU data protection legislation, as the dataset contains personal data that could potentially, using the existing study database, be linked to an identifiable living natural person. Ten years after the last publication, identifying information will be deleted, and anonymized IPD together with trial documentation will be archived for long-term storage. From that time onward, anonymized IPD will be available from the corresponding author upon reasonable request.
