# Correction: Basic emotions reported by individuals with persistent physical symptoms receiving exposure therapy versus healthy lifestyle promotion in primary care

**DOI:** 10.1038/s41598-026-57897-1

**Published:** 2026-06-17

**Authors:** Jonna Hybelius, Sandra af Winklerfelt Hammarberg, Sigrid Salomonsson, Caroline Wachtler, Majken Epstein, Anna Olsson, Emma Strand, Lina Söderström Winter, Alice Ahnlund Hoffmann, Edward Spansk, Tomas Åkerlund, Daniel Björkander, Amanda Kosic, Gabriel Chahin, John Wallert, Eva Toth-Pal, Steven Nordin, Michael Witthöft, Erland Axelsson

**Affiliations:** 1 Division of Family Medicine and Primary Care, Department of Neurobiology, Care Sciences and Society, Karolinska Institutet, Huddinge, Sweden; 2 Liljeholmen University Primary Health Care Center, Academic Primary Health Care Center, Region Stockholm, Stockholm, Sweden; 3 Centre for Psychiatry Research, Department of Clinical Neuroscience, Karolinska Institutet, & Stockholm Health Care Services, Region Stockholm, Stockholm, Sweden; 4 Division of Psychology, Department of Clinical Neuroscience, Karolinska Institutet, Stockholm, Sweden; 5 Meliva Primary Health Care Center Kungshörnet, Uppsala, Sweden; 6 School of Law, Psychology and Social Work, Örebro University, Örebro, Sweden; 7 Department of Psychology, Umeå University, Umeå, Sweden; 8 Department of Clinical Psychology and Psychotherapy, Ruhr University Bochum, Bochum, Germany

Correction to: *Scientific Reports* 10.1038/s41598-026-39962-x, published online 17 February 2026

The original version of this Article contained an error. In the Results section under the subheading ‘Test–retest reliability and construct validity of emotions questionnaire’, the incorrect test–retest reliability estimates for the emotion surprise were published and therefore do not match the correct estimates published in Table DS1 in the Supplementary Information file which accompanies the article. In addition, the estimates for shame were inadvertently omitted from the reported ranges summarizing the lowest and highest test–retest reliability estimates across the remaining emotions.

As a result,

“In the PPS participant data, the test–retest reliability for the emotions was acceptable (over a mean of 6.5 days [SD = 3.6]; *r*s = 0.60–0.88, *ρ* = 0.60–0.83, ICCs = 0.68–0.80), but with contradictory indicators for surprise, where the rank correlation was suboptimal despite a promising ICC (*r* = 0.67, *ρ* = 0.46, ICC = 0.81). We therefore refrained from further analysis of the surprise item.”

now reads:

“In the PPS participant data, the test–retest reliability for the emotions was acceptable (over a mean of 6.5 days [SD = 3.6]; *rs* = 0.60–0.88, *ρs* = 0.46–0.83, ICCs = 0.68–0.81), with the exception of surprise (*r* = 0.07, *ρ* = 0.45, ICC = 0.60). We therefore refrained from further analysis of the surprise item, although the interpretation of these findings is complicated by the possibility that low test–retest reliability could partly reflect that surprise may be inherently less stable across time points than the other emotions.”

The original Article has been corrected.

