# Supplementary material for: Basic emotions reported by individuals with persistent physical symptoms receiving exposure therapy versus healthy lifestyle promotion in primary care
Source: Sci Rep. 2026 Feb 17;16:7170. doi: 10.1038/s41598-026-39962-x (PMC12920773; doi:10.1038/s41598-026-39962-x)
Supplement: Supplementary file 1 — Supplementary Information. [file 41598_2026_39962_MOESM1_ESM.pdf]

Supplement for:

Basic emotions reported by individuals with persistent physical symptoms receiving exposure therapy versus healthy lifestyle promotion in primary care

Study-specific Basic Emotions Questionnaire........ 2

Table DS1. Test-retest reliability of the basic emotions questionnaire in a subsample (n=20) of patients with persistent physical symptoms who completed the pre-treatment assessment within 14 days from screening 3

Table DS2. Test-retest reliability in the healthy volunteers subsample (n=160) over 14 days 4

Table DS3. Pearson Correlations between basic emotions items concerning fear and sadness versus previously validated self-rating scales for evaluation of construct validity in the sample of patients with persistent physical symptoms (n=159) 5

Table DS4. Intercorrelations between basic emotions among patients with persistent physical symptoms (n=159)........ 6

Table DS5. Pearson correlations between basic emotions and specific somatic symptom domains among patients with persistent physical symptoms (n=159) 7

Table DS6. Moderator analyses of mean change in internet-delivered exposure therapy vs healthy lifestyle promotion over 10 weeks........ 8

References........ 10

Study-specific Basic Emotions Questionnaire

Emotions related to bodily symptoms

The following questions concern emotions related to your bodily symptoms. By *bodily symptoms*, we mean, for example, pain, stomach or digestive problems, heart palpitations, changes in breathing, urinary discomfort, menstrual cramps, reduced sexual function, dizziness, nausea, changes in vision, changes in smell, changes in taste, changes in hearing, a lump in the throat, sweating, eczema, itch, trembling, muscle twitches, unsteadiness, or fatigue.

How have you felt about your bodily symptoms during **the past week**?

Surprise: Your symptoms made you feel surprised.

No, not at all true ____ Yes, completely true

Joy: Your symptoms made you happy.

No, not at all true ____ Yes, completely true

Anger: Your symptoms made you angry.

No, not at all true ____ Yes, completely true

Fear: Your symptoms made you feel afraid.

No, not at all true ____ Yes, completely true

Shame: You felt ashamed of your symptoms.

No, not at all true ____ Yes, completely true

Sadness: Your symptoms made you sad.

No, not at all true ____ Yes, completely true

Disgust: You felt disgusted by your symptoms.

No, not at all true ____ Yes, completely true

Table DS1. Test-retest reliability of the basic emotions questionnaire in a subsample (n=20) of patients with persistent physical symptoms who completed the pre-treatment assessment within 14 days from screening.

	**Pearson *r***	**Spearman rho**	**ICC**
**Basic emotions questionnaire**			
Anger	0.71	0.62	0.70
Disgust	0.88	0.83	0.80
Fear	0.60	0.68	0.70
Joy	0.66	0.60	0.68
Sadness	0.73	0.65	0.69
Shame	0.67	0.46	0.81
Surprise	0.07	0.45	0.60
**Conventional symptom scales**			
PHQ-2	0.67	0.78	0.78

Notes. Test-retest reliability was assessed using Pearson correlations, Spearman correlations, and two-way random effects absolute agreement intraclass correlation coefficients (ICC). To allow for post hoc estimation of test-retest reliability for the basic emotions questionnaire in the clinical subsample, a subset of patients who completed the pre-treatment assessment within 14 days of filling out the screening questionnaires was selected. However, the basic emotions questionnaire administered as part of the screening procedure concerned the experience of basic emotions related to somatic symptoms over the previous month, while the basic emotions questionnaire administered at the pre-treatment assessment concerned the past week. For comparison, the test-retest reliability of a previously validated conventional depression screening questionnaire was also estimated, i.e., the PHQ-2, the Patient Health Questionnaire 2 (the first two items on the Patient Health Questionnaire 9, investigating core depression symptoms).

Table DS2. Test-retest reliability in the healthy volunteers subsample (n=160) over 14 days

	**Pearson *r***	**Spearman rho**	**ICC**
**Basic emotions questionnaire[a]**			
Anger	0.49	0.42	0.64
Disgust	0.26	0.42	0.37
Fear	0.36	0.32	0.51
Joy	0.20	0.36	0.33
Sadness	0.34	0.45	0.50
Shame	0.59	0.49	0.73
Surprise	0.28	0.48	0.43
**Conventional symptom scales**			
GAD-7	0.52	0.56	0.52
HAI-14	0.75	0.71	0.73
PHQ-2	0.56	0.35	0.56

Notes. Test-retest reliability of the basic emotions questionnaire was assessed using Pearson correlations, Spearman correlations, and two-way random effects absolute agreement intraclass correlation coefficients (ICC). For comparison, test-retest reliability was also assessed in the same way in previously validated measures of anxiety and depression. GAD-7, the general anxiety questionnaire; HAI-14, the 14 item health anxiety inventory; PHQ-2, the Patient Health Questionnaire 2 (the first two items on the Patient Health Questionnaire 9, investigating core depression symptoms).

[a] Basic emotions related to physical symptoms over the past week, measured by a study-specific questionnaire.

Table DS3. Pearson Correlations between basic emotions items concerning fear and sadness versus previously validated self-rating scales for evaluation of construct validity in the sample of patients with persistent physical symptoms (n=159)

	**Fear**	**Sadness**
	r	***r***
**GAD-7**		
Sum score	0.39***	N/A
**HAI-14**		
Sum score	0.41***	N/A
**PHQ-2**		
Sum score	N/A	0.34***

Notes. For construct validity, correlations between the items on the basic emotions questionnaire (weekly version) concerning fear and sadness were estimated versus previously validated measures of anxiety and depression. GAD-7, the general anxiety questionnaire; HAI-14, the 14 item health anxiety inventory; N/A, not applicable/not estimated; PHQ-2, the Patient Health Questionnaire 2 (the first two items on the Patient Health Questionnaire 9, investigating core depression symptoms).
[a] Basic emotions related to physical symptoms over the last week, measured by a study-specific questionnaire.
*** $p < 0.001$.

Table DS4. Intercorrelations between basic emotions among patients with persistent physical symptoms (n=159)

	**Anger**	**Disgust**	**Fear**	**Joy**	**Sadness**	**Shame**	**Surprise**
	r	***r***	***r***	***r***	***r***	***r***	***r***
**Anger**	1.00	0.26	0.31	0.07	0.41	0.28	0.14
**Disgust**		1.00	0.24	0.06	0.33	0.66	0.19
**Fear**			1.00	0.05	0.44	0.40	0.33
**Joy**				1.00	-0.04	0.08	0.16
**Sadness**					1.00	0.43	0.17
**Shame**						1.00	0.26
**Surprise**							1.00

Notes. According to commonly applied thresholds for assessment of collinearity, the basic emotions were considered independent, as all intercorrelations were below 0.70 (r = 0.24-0.66)[1].
[a] Basic emotions related to physical symptoms over the last week, measured by a study-specific questionnaire.

Table DS5. Pearson correlations between basic emotions[a] and specific somatic symptom domains among patients with persistent physical symptoms (n=159)

	**Anger**	**Disgust**	**Fear**	**Joy**	**Sadness**	**Shame**
	r	***r***	***r***	***r***	***r***	***r***
**PHQ-15 subscales**						
Cardiopulmonary symptoms	0.03	0.06	0.23*	0.04	0.27*	0.13
Fatigue symptoms	0.13	-0.01	0.16	0.13	0.14	0.07
Gastrointestinal symptoms	0.02	0.32*	0.01	0.05	0.03	0.24*
Pain symptoms	0.10	-0.11	0.06	0.03	0.16	-0.04

Notes. All intercorrelations between the basic emotions were below 0.70 (r = 0.24-0.66; see Table DS4). PHQ-15, Patient Health Questionnaire 15 (conventional 4-week version; sum score 0-30, domain subscales measuring cardiopulmonary, fatigue, gastrointestinal, and pain symptoms are scored 0-2).

[a] Basic emotions related to physical symptoms over the last week, measured by a study-specific questionnaire.

* Significance determined after adjusting for multiple testing using the Benjamini-Hochberg procedure to control the false discovery rate (FDR)[2].

Table DS6. Moderator analyses of mean change in internet-delivered exposure therapy vs healthy lifestyle promotion over 10 weeks

Potential moderator	Treatment	Within-group moderation in pre-post change				Moderation of treatment difference in pre-post change			
		PHQ-15w		WD2-12		PHQ-15		WD2-12	
		est (95% CI); *z*	*p*	est (95% CI); *z*	*p*	est (95% CI); *z*	*p*	est (95% CI); *z*	*p*
Basic emotions[a]									
Anger	Exposure	-0.18 (-0.52, 0.17); -1.01	0.313	-0.22 (-1.09, 0.65); -0.49	0.622	-0.02 (-0.47, 0.43); -0.08	0.933	-0.07 (-1.23, 1.09); -0.11	0.910
	HLP	-0.16 (-0.45, 0.14); -1.06	0.290	-0.15 (-0.91, 0-61); -0.39	0.693				
Disgust	Exposure	-0.48 (-0.86, -0.10); -2.49	0.014[b]	0.15 (-0.82, 1.13); 0.30	0.761	-0.61 (-1.10, -0.13); -2.48	0.014[b]	0.12 (-1.14, 1.38); 0.19	0.850
	HLP	0.13 (-0.17, 0.44); 0.88	0.382	0.03 (-0.77, 0.82); 0.07	0.942				
Fear	Exposure	-0.30 (-0.66, 0.07); -1.61	0.108	0.22 (-0.68, 1.12); 0.48	0.632	-0.39 (-0.88, 0.10); -1.56	0.121	0.54 (-0.70, 1.77); 0.85	0.394
	HLP	0.09 (-0.24, 0.42); 0.55	0.586	-0.32 (-1.17, 0.53); -0.74	0.461				
Joy	Exposure	0.40 (-1.56, 2.35); 0.40	0.690	-1.12 (-6.16, 3.93); -0.43	0.664	0.70 (-1.89, 3.28); 0.53	0.596	-0.07 (-6.64, 6.51); -0.02	0.984
	HLP	-0.30 (-2.00, 1.40); -0.35	0.729	-1.05 (-5.24, 3.14); -0.49	0.622				
Sadness	Exposure	-0.37 (-0.70, -0.04); -2.21	0.028[b]	-0.65 (-1.47, 0.16); -1.58	0.116	-0.39 (-0.83, 0.05); -1.76	0.080	-0.63 (-1.74, 0.47); -1.13	0.260
	HLP	0.02 (-0.27, 0.31); 0.14	0.888	-0.02 (-0.77, 0.73); -0.05	0.960				
Shame	Exposure	-0.23 (-0.53, 0.08); -1.45	0.148	0.11 (-0.67, 0.88); 0.28	0.781	-0.09 (-0.52, 0.35); -0.40	0.691	0.55 (-0.57, 1.66); 0.97	0.335
	HLP	-0.14 (-0.45, 0.17); -0.87	0.384	-0.44 (-1.24, 0.36); -1.08	0.283				

Potential moderator	Treatment	Within-group moderation in pre-post change		Moderation of treatment difference in pre-post change	
		Fear[a]		Fear[a]	
		est (95% CI); *z*	*p*	est (95% CI); *z*	*p*
Fear[a]	Exposure	-0.48 (-0.64, -0.32); -5.90	0.000***	0.02 (-0.19, 0.24); -0.20	0.839

	HLP	-0.50 (-0.65, -0.35); -6.71	0.000***

Notes. Estimates derived from linear mixed effects regression models fitted on multiply imputed data, and moderation of the treatment difference in pre-post change was tested on the basis of the coefficient for the three-way interaction of time, condition, and potential moderator. According to commonly applied thresholds for assessment of collinearity, the basic emotions were considered independent, as all intercorrelations were below 0.70 (r = 0.24-0.66) [1]. All scales were administered as self-report questionnaires via the Internet at the pretreatment assessment (pre) and the post-treatment assessment (post), and the PHQ-15w was also administered each week over the 10-week treatment phase. HLP, Internet-delivered healthy lifestyle promotion; PHQ-15, Patient Health Questionnaire 15 rephrased to concern the past week only; WD2-12, 12-item self-report World Health Organization Disability Assessment Schedule 2 (scored 0-100).

[a] Basic emotions related to physical symptoms over the past week, measured by a study-specific questionnaire.

[b] Not significant after adjusting for multiple testing using the Benjamini-Hochberg procedure to control the false discovery rate (FDR)[2].

*** p < 0.001 Not adjusted for multiple testing due to pre-specified, directed hypothesis.

References

1. Dormann, C. F. et al. Collinearity: a review of methods to deal with it and a simulation study evaluating their performance. *Ecography* **36,** 27–46 (2013).
2. Benjamini, Y. & Hochberg, Y. Controlling the False Discovery Rate: A Practical and Powerful Approach to Multiple Testing. *J. R. Stat. Soc. Ser. B Methodol.* **57,** 289–300 (1995).
